# Study protocol: randomized controlled trial of an individualized music intervention for people with dementia in the home care setting

**DOI:** 10.1186/s12888-024-05697-0

**Published:** 2024-03-26

**Authors:** Elisabeth Jakob, Juliane Meininger, Mareike Hillebrand, Lisette Weise, Gabriele Wilz

**Affiliations:** Department of Counseling and Clinical Intervention, Institute of Psychology, Friedrich Schiller University Jena, Humboldtstrasse 11, 07743 Jena, Germany

**Keywords:** Non-pharmacological intervention, Receptive music therapy, Alzheimer’s disease, Behavioural and psychological symptoms of dementia, Neuropsychiatric symptoms, Quality of life, Family caregivers

## Abstract

**Background:**

Studies suggest that *individualized music listening* is an effective, non-pharmacological intervention for improving the quality of life of people with dementia in the institutional care setting. Noting that most people with dementia live at home, we conduct a randomized controlled trial to assess the feasibility and effectiveness of an app-based individualized music listening intervention for people with dementia in the home care setting. The intervention is delivered by family caregivers.

**Methods:**

We will recruit *N* = 130 dyads consisting of one person with dementia living at home and their family caregiver. After a baseline assessment, dyads are randomly assigned by gender to either the intervention or control group. People with dementia in the intervention group listen to individualized music playlists for 20 min every other day for six weeks via the self-developed *Individualized Music and Dementia* app. The control group receives standard care. All dyads complete paper-and-pencil questionnaires six weeks before the start of the intervention (T0), directly before the intervention (T1), directly after the intervention (T2), and six weeks later (T3). During the intervention period, all caregivers also complete daily ecological momentary assessments via the app. During three home visits, a trained project member will observe the dyads and collect hair samples. After the intervention, semi-structured interviews will be conducted to collect information about participants’ experiences with the app and intervention. The primary outcome is the attainment of individual goals established during the baseline assessment. Secondary outcomes are the well-being, physiological stress and quality of life of people with dementia and their caregivers; people with dementia’s behavioural and psychological symptoms of dementia, resistance during care, and reactions to the music; caregivers’ burden of care, positive aspects of care, and caregiving self-efficacy; and the quality of the caregiver-care recipient interaction.

**Discussion:**

Our study will assess the extent to which an app-based individualized music listening intervention is feasible and effective for enhancing the well-being and quality of life of people with dementia living at home and their family caregivers.

**Trial registration:**

German Clinical Trials Register DRKS00025502 and ISRCTN registry ISRCTN68084105, https://doi.org/10.1186/ISRCTN68084105

**Supplementary Information:**

The online version contains supplementary material available at 10.1186/s12888-024-05697-0.

## Background

Around 55 million people worldwide are currently living with dementia, and this number is expected to dramatically increase in the future as populations grow older [1]. People with dementia increasingly need assistance and support as their disease progresses. Most people with dementia live at home, and care is most often provided by a family member or other informal caregiver [2, 3]. There is thus an urgent need for interventions – especially non-pharmacological interventions [4, 5] – that improve the quality of life of people with dementia and their caregivers that can also feasibly be implemented in the home care setting. A particularly important target for intervention is decreasing the behavioural and psychological symptoms of dementia (BPSD) such as agitation, anxiety or depressive symptoms, which significantly reduce the well-being and quality of life of people with dementia [6] and their caregivers [7, 8], as well as negatively impact the quality of interactions between the caregiver and care recipient [9, 10].

*Individualized music listening* (IML) – that is, listening to personally-meaningful music based on one’s own preferences and experiences – has been shown to be an effective non-pharmacological intervention for enhancing the quality of life of people with dementia in the institutional care setting [11]. The use of IML interventions is supported by neuroscientific evidence, which shows that the brain regions associated with long-term musical memory are relatively unaffected by dementia [12]. Thus, people with dementia remember personally-meaningful music very well. Although the precise mechanisms are still unclear, recent reviews of studies conducted in the institutional care setting have concluded that IML interventions have considerable potential for improving the well-being, social behaviour and BPSD (particularly agitation) of people with dementia [13–16]. A very recent randomized controlled trial (RCT) showed that IML reduced agitation, aggression, and disorientation [17], and helped people with dementia accomplish their own, individual goals (e.g., regarding the people with dementia’s social participation and/or mood) [18]. However, reviews have also emphasized that the effects of IML interventions are highly heterogeneous across studies, and more methodologically rigorous studies and large-scale RCTs are urgently needed [13–16].

A further caveat in research on IML is that the vast majority of research on IML interventions has taken place in the institutional care setting (e.g., nursing homes). Thus, the feasibility and effectiveness of IML interventions in the home care setting is uncertain. Differences in, for instance, the characteristics of the care recipients (e.g., dementia severity) or the physical living space of private and institutional settings (e.g., comfort, available distractions) may affect the feasibility and effectiveness of IML interventions. Nevertheless, a handful of quasi-experimental studies provide first evidence that IML delivered in the home care setting effectively reduce distress [19, 20], agitation [21] and pain levels [22] among people with dementia. There is also some evidence that home-based IML interventions benefit caregivers by increasing caregiver self-efficacy [19] or providing caregivers with an opportunity to take a break [20].

Building on existing evidence of the effectiveness of IML in the institutional and, to a much lesser extent, the home care setting, we conduct the first RCT to test the feasibility and effectiveness of a self-developed, app-based IML intervention for people with dementia living at home. The intervention is delivered by family caregivers in the people with dementia’s own home environment. Most existing studies have relied on professional caregivers and/or music therapists to implement the IML intervention. Designing interventions that can be delivered by family caregivers is key to making it possible to implement IML interventions on a wider scale. First evidence suggests that family caregivers can indeed successfully deliver IML interventions in the home care setting [23], although the intervention must be adapted to the caregivers’ technological skills and experience [19, 20].

We expect that our app-based IML intervention can provide a cost-effective, highly acceptable intervention to improve the quality of life and well-being of people with dementia and their caregivers. Previous research has indicated high heterogeneity in how people with dementia react to IML [24]. We therefore focus on *individual* goal attainment as our primary outcome. We additionally examine the effects of the intervention on caregivers and people with dementia’s well-being, BPSD, the caregiving experience, and the quality of caregiver-care recipient interactions.

We also add to the literature by investigating the impact of IML on the physiological stress of caregivers and people with dementia. Although it is plausible that IML benefits people with dementia at least partly by reducing physiological stress, so far few empirical studies have examined how IML impacts indicators of physiological stress (e.g., heart rate, blood pressure). Moreover, most existing studies have focused exclusively on immediate and short-term effects [24]. Existing evidence has been somewhat mixed, with preliminary findings suggesting potential autonomic down-regulation following IML in the short-term (for a review, see Sittler et al., [25]). In our study, we examine the short- and longer-term impact of IML on heart rate variability and, for the first time, cortisol levels in hair. Analysing cortisol in the hair of people with dementia is feasible and also has a number of advantages in pragmatic RCTs [26], and offers a promising methodological approach for investigating the longer-term effects of IML on physiological stress [26].

## Methods

### Design

**Fig. 1:**
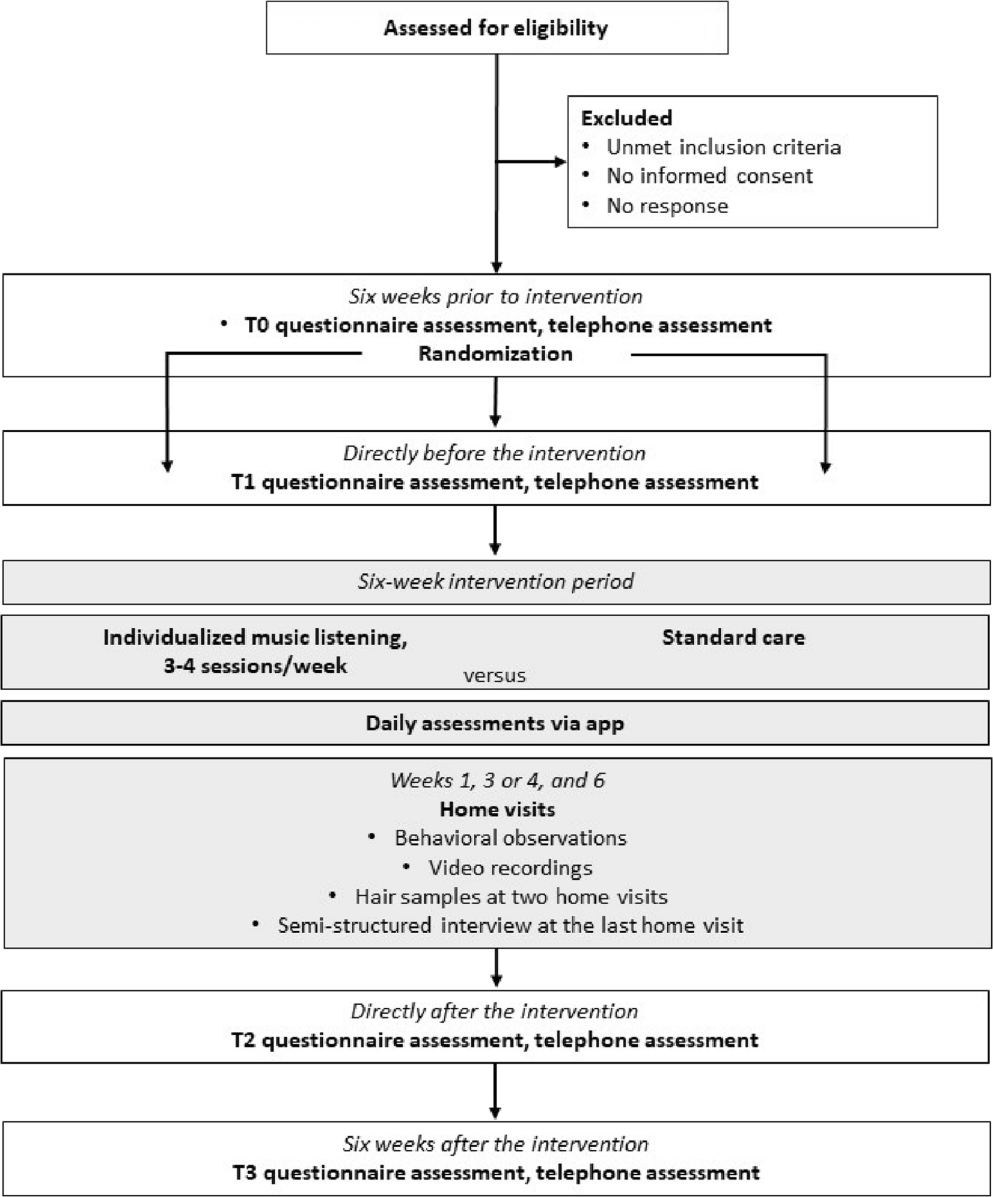
Overview of the design of the study

We conduct an RCT with an intervention and a control group. All participants complete paper-and-pencil questionnaires at four assessment points: baseline (T0; six weeks before the intervention), pre-test (T1; immediately before the intervention), post-test (T2; immediately after the intervention) and follow-up (T3; six weeks after the intervention). During the six-week intervention period, all caregivers additionally complete daily assessments (i.e., ecological momentary assessments) and three home visits take place. During the home visits, a project member conducts behavioural observations and collects hair samples. Provided consent, video recordings will be made. At the last home visit, a project member conducts semi-structured interviews. Figure 1 provides an overview of the design.

### Participants

The RCT is based on dyads consisting of one person with dementia living at home and their family caregiver. To participate in the study, the person with dementia must have medically-diagnosed dementia, be living at home, and have a health care proxy. Exclusion criteria for people with dementia are severe hearing impairment or planning to move to the institutional care setting within the next three months. No restriction is made regarding the age of the participating people with dementia. Exclusion criteria for caregivers are major physical health impairments or progressive illness, psychiatric diagnosis, obvious cognitive impairment, difficulty understanding German, or participation in another study involving caregivers.

### Recruitment

Dyads will be mainly recruited in Thuringia and adjacent regions in Germany. With the assistance of regional cooperation partners (e.g., medical practices; the German Alzheimer Society of Thuringia), we will recruit participants via posters and flyers; online, print and radio media; and project presentations at planned events and support groups for caregivers. Interested caregivers can contact the project team by telephone during office hours, personally at project presentations, via the contact form on the project website at www.musik-demenz.de, or by e-mail. Potential participants will receive detailed information about the study. After checking that the inclusion criteria have been met, the research team will mail potential participants written information about the study and data protection, a consent form and a stamped return envelope. The text will be formulated for caregivers and in simple language for people with dementia. Dyads who have given informed consent form will be included in the study.

### Sample size

Based on prior research on the effects of IML on people with dementia in the institutional care setting [18, 24, 27], we expect that IML will have small to medium effect sizes (f = 0.2) on our outcome variables. Given our two-group design, an α of 0.05, and a power (1 − β) of 0.95, a sample of *N* = 98 dyads will be needed in order to reliably detect effects. Drop out in studies with a similar target group has ranged from 18% [18] to 36% [28]. To account for drop out, we therefore aim to recruit *N* = 130 dyads.

### Procedure

#### Baseline assessment

All caregivers will receive a paper-and-pencil questionnaire per mail with a stamped return envelope. The questionnaire will assess sociodemographic and other control variables (e.g., sleep quality, physical complaints), as well as the caregiver’s well-being and perceptions of the caregiving experience. Caregivers will also provide information about their perceptions of the well-being and BPSD of the person with dementia, and the quality of the caregiver-care recipient interactions. A research assistant will contact the caregiver by telephone to debrief the completed questionnaire and ask caregivers about depressive symptoms of the person with dementia. Caregivers will also be asked to formulate at least one goal for the person with dementia to accomplish during the six-week intervention period. Caregivers will describe observable criteria by which accomplishment of the goal can be determined.

#### Randomization

Following the baseline assessment, a research assistant uninvolved in the assessments or implementation of the study will use a computer-generated randomization list from www.random.org to randomly assign participants by gender to either the intervention (IG) or control group (CG). A research assistant will contact the dyads by telephone to inform them about their allocation and schedule a first home visit. It will not be possible to blind participants to their treatment allocation due to the nature of the intervention.

#### The individualized music and dementia app

All dyads will receive a tablet (Samsung Galaxy Tab A8) pre-installed with the self-developed Individualized Music and Dementia (IMuD) app. We designed the IMuD-App to be exceptionally user-friendly (e.g., large font, explanations in simple language, intuitive navigation). The IML intervention is delivered via the app. All caregivers will additionally use the app to complete and submit daily assessments as described below. Throughout the intervention, caregivers can also use the app to contact the research team using a contact form.

#### Intervention

After randomisation, the tablet will be sent to participants of the IG via mail. Caregivers in the IG will complete an assessment of music that is personally-relevant to the person with dementia. The assessment includes open question (e.g. “Which songs were heard at family celebrations?”), multiple-choice questions (e.g. regarding the preferred genre) and, based on a search of popular music from different decades, a list of examples of popular artists and song titles. To the extent possible, the assessment will be completed together with the person with dementia. The caregiver will submit the completed assessment via the app. Based on the completed assessment, the project team will create three individualized music playlists for each person with dementia in the IG.

People with dementia in the IG will then listen to their individualized playlists for 20 min every other day (3–4 times a week) for six weeks, through headphones if possible. Dyads can choose when the sessions take place and skip a session if necessary. The caregiver and/or person with dementia can freely choose one of the three music playlists which are stored on and played through the app. The caregiver will be supported to find out what accompaniment the person with dementia needs when listening to the music. Videos and written recommendations in the app provide guidance on how the caregiver can optimize the listening situation, interact with the person with dementia during the listening sessions, and respond to any challenging situations (e.g., lower the volume or change the song if the person with dementia becomes agitated).

Participants in the CG will receive no IML intervention and organize their everyday life as usual. Members of the CG will receive 200 Euro at the end of successful participation in the study. The renumeration is provided to the dyad as a unit. Dyads of the IG will receive no renumeration.

The project team will contact participants in both the IG and the CG regularly in order to encourage continued participation and address any arising problems.

#### Home visits

During the six-week intervention period, two project members will conduct a total of three home visits per dyad. The home visits will take place in the first, third or fourth (depending on the dyad’s availability) and sixth week of the intervention period. The dyads will receive informational brochures on dementia during each home visit.

During the first home visit, caregivers in the CG will receive the same tablet as caregivers in the IG. The project member will provide the caregiver with a detailed technical briefing of the app and an introduction to the daily assessments. Together with the project member, caregivers in the IG will additionally watch instructional videos via the app regarding how to conduct the music listening sessions.

The project member will then use the Mini-Mental-State-Examination (MMSE) [29] to assess the severity of dementia of the care recipient. If the MMSE score exceeds 10, the project member will use the Subjective Quality of Life Inventory [30] to assess the person with dementia’s ability to make statements about their own well-being.

During each home visit, a trained project member will use a revised version of the Dementia Coding System (DeCS) [17] to record the reactions and behaviours of the person with dementia. The coding system was adapted for the current project and includes 26 items covering the people with dementia’s emotional, physical, and communicative behaviour (e.g., facial expressions, gestures, body movement). A trained research assistant will use the DeCs to assess the behaviour of the person with dementia in 15 time-units of four minutes each (60 min in total). For participants in the IG, the project member will record the behaviour of the person with dementia 20 min before, 20 min during, and 20 min after the listening session. For participants in the CG, the project member will record the behaviour of the person with dementia during a 60-min open conversation.

Provided consent, the behavioural observations will be filmed. One camera will record the face of the caregiver, a second camera will record the face of the person with dementia, and a third camera will record a 360° view of the room. As described below, the videos will be analysed for indications of (acute) physiological stress, reactions to the music and dyadic interactions at a later time point.

Provided consent, the research team will additionally collect hair samples from the person with dementia and the caregiver during the first and third home visits. As described below, the hair samples will be analysed for indications of chronic physiological stress.

During the third home visit, a project member will conduct a semi-structured interview to capture participants’ experiences with the tablet, app, daily assessments, home visits and project team. Caregivers in the IG will also be asked to describe their perceptions of the feasibility, acceptance, applicability, and effects of the IML intervention. To the extent possible, the participants with dementia will be asked to describe their own experiences.

The research team will collect the tablets and further technology at the end of the third home visit.

#### App-based daily assessments

During the six-week intervention period, all caregivers will use the app to complete daily ecological momentary assessments of their own well-being and arousal, the well-being and arousal of the people with dementia and the caregiver-care recipient interaction. At a time of the caregivers’ choosing, the app will prompt the caregiver to complete the questionnaire with an audible beep. Caregivers can skip an assessment if they do not have time on a day.

#### Questionnaire assessments

In addition to the app-based daily assessments and the home visits, paper-and-pencil questionnaires will be sent to the participants by mail directly before the intervention (T1), directly after the intervention (T2), and six weeks later (T3). All questionnaire assessments are identical.

#### Adverse events

Caregivers will be supported to find out what accompaniment the person with dementia needs when listening to music. Caregivers should remain present during the first IML sessions to be able to intervene if necessary. To empower the caregivers, project members will personally provide a detailed introduction to the technology and the IML intervention. If the person with dementia repeatedly reacts negatively to the music, it is possible to change the song permanently, lower the volume, pause or stop the music. Participants can stop a listening session or drop out of the study at any time.

### Outcome measures

An overview of the assessments and outcome variables is provided in additional file 1.

#### Primary outcome: individual goal attainment

Standardized outcome measures may underestimate the effects of IML. We therefore focus on participants’ *individual goal attainment* after six weeks of IML or treatment as usual as the primary outcome. We will use the Goal Attainment Scaling (GAS) approach to assess individual goal attainment [31]. The extent to which the individual goals formulated during the T0 telephone interview were attained will be quantified using a 5-point scale (-2 = “severe deterioration”; + 2 = “complete goal attainment”; [32]).

#### Well-being of person with dementia: self and caregiver perceptions

Based on the circumplex model of emotion [33], caregivers will use visual analogue scales ranging from 0 to 100 [34] to assess the person with dementia’s *emotional well-being* and *arousal* daily via the app and as part of the paper-and-pencil questionnaires assessments at T0, T1, T2, and T3.

To the extent possible, participants with dementia will provide self-reports of their well-being and quality of life. During each of the three home visits, participants with dementia will use the Dementia Mood Picture Test [35] to self-assess their *emotional well-being* by selecting which of six faces (bad mood, angry, sad, worried, good mood, happy) best represents their emotional state. Participants with dementia will also use the Heidelberg Instrument for the Quality of Life of Dementia Patients [36] to indicate their *overall life satisfaction* (1 = “not at all satisfied” to 4 = “very satisfied”).

#### Behavioural and psychological symptoms of dementia

Caregivers will assess the person with dementia’s BPSD as part of the paper-and-pencil questionnaires assessments at T0, T1, T2, and T3. Caregivers will use the German-version of the Behavioural Pathology in Alzheimer’s Disease Rating Scale [25]. Caregivers use a dichotomous response scale (0 = “not present”, 1 = “present”) to indicate the extent to which the people with dementia displays each of 23 BPSD covering four factors (Paranoid and Aggressiveness, Hallucinations and Agitation, Affective Disturbances, Anxieties and Phobia). Caregivers will additionally use the Cornell Scale for Depression in Dementia [37] to assess the people with dementia’s *depressive symptoms*. Specifically, caregivers are asked to indicate the extent to which the person with dementia has each of 19 symptoms of depression using four answer categories (0 = “absent”, 1 = “mild/intermittent”, 2 = “severe”, 3 = “assessment not possible”). Caregivers will also use a visual analogue scale ranging from 0 to 100 to assess the people with dementia’s *resistance during care *[38].

During the home visits, a trained project member will also use the revised version of DeCS [17] to assess the person with dementia’s BPSD as part of the behavioural observations. A subset of 14 of the 26 behaviours assessed by the revised DeCS are BPSD (e.g., resistance/refusal behaviours).

#### Caregiver’s well-being

Caregivers will assess their *emotional well-being* and *arousal* daily via the app (daily assessments as well as directly before and after IML) and as part of the pencil-and-paper assessments at T0, T1, T2, and T3. Caregivers use visual analogue scales ranging from 0 to 100 [34].

#### The caregiving experience

Caregivers will assess the caregiving experience as part of the paper-and-pencil questionnaires at T0, T1, T2, and T3. To assess *caregiving burden*, caregivers will complete the German short version of the Home Care Scale (10 items, 0 = “exactly correct” to 3 = “not correct”; [39]). To assess *positive aspects of caregiving*, caregivers complete a German version of the Positive Aspects of Care Questionnaire (6 items; 0 = “strongly disagree” to 4 = “strongly agree”; [40]). The German version of the questionnaire is currently being validated. To assess caregivers’ *caregiving self-efficacy*, caregivers will complete the Satisfaction with One's Own Performance as a Caregiver subscale of the abbreviated Sense of Competence Questionnaire (5 items, 0 = “does not apply” to 5 = “applies”; [41]).

#### Immediate reactions to individualized music listening

Directly before and after IML, participants with dementia in the IG group will use the Smiley Assessment Scale (SAS) to assess their *current mood* via the app. Participants with dementia select which of three faces (happy, neutral, unhappy) best depict their mood (adapted from Rosenberg and Mittelman & Epstein [42, 43]).

Directly before and after IML, caregivers in the IG group will use visual analogue scales ranging from 0 to 100 [34] to assess the person with dementia’s *emotional well-being* and *arousal* via the app. Directly after the listening session, caregivers will additionally use the app to indicate whether the person with dementia displayed each of 19 behaviours (e.g., “singing”, “listening attentively”; yes/no) during the listening session. In addition, caregivers can describe their observations of the person with dementia during the listening session using an open answer format.

During the three home visits, a trained research assistant will use a revised version of the DeCS [17] to assess the behaviour of the person with dementia during the listening session.

#### Dyadic interaction quality

Caregivers will assess the *dyadic interaction quality* daily via the app and as part of the paper-and-pencil questionnaire at T0, T1, T2, and T3. Caregivers will use a self-constructed questionnaire (8 items, e.g., “We experience moments of joy together”; 0 = “strongly disagree” to 4 = “strongly agree”).

After each home visit, a project member will answer a single item on the dyad’s *global relationship quality* (-2 = “very bad” to + 2 = “very good”). The project member will also complete a self-constructed *relationship quality questionnaire* (16 items; e.g., “They get along well with each other”; -2 = “completely disagree” to + 2 = “completely agree”, or “assessment not possible”).

Trained raters will additionally use videos of the home visits to assess the quality of the dyadic interaction. Three camera recordings are made: frontal view of the person with dementia, frontal view of the caregiver and recording of the overall setting.

#### Acute and chronic physiological stress

We will use videos of the home visits and FaceReader software (Noldus) to assess the *heart rate variability* of the person with dementia and the caregiver as an indicator of acute physiological stress before, during, and shortly after IML (immediate and short-term effects) [26, 44]. FaceReader uses photoplethysmography to detect heart rate in video recordings of faces [45]. We will use the *cortisol/dehydroepiandrosterone concentrations* in the hair samples collected in the first and third home visits as an indicator of chronic physiological stress before and after six weeks of IML (i.e., longer-term effects) [26, 46].

#### Intervention experiences, fidelity and acceptance

We use semi-structured interviews to capture participants’ *qualitative experiences* with the study and intervention. In addition, the app records the frequency, duration, pause or end of music listening as well as whether songs are skipped. We will use this quantitative information to assess the *quality of the playlists* as well as *intervention fidelity* (i.e., the extent to which the intervention was implemented as intended). All caregivers will use the System Usability Scale to rate *the user-friendliness of the app and tablet* (10 items; 1 = “do not agree at all” to 5 = “fully agree”; [47]) during the third home visit. Caregivers in the IG will additionally complete a self-developed paper-and-pencil questionnaire to assess their *overall experience* with the app, tablet, and intervention (14 items, e.g., “Would you want the person with dementia to continue with the listening sessions?”, 1 = “definitely not” to 4 = “definitely yes”).

### Statistical analysis

We will use SPSS (IBM Corp., Armonk, NY) and R (R Development Core Team, Vienna, Austria) to conduct all analyses. We will check whether there are meaningful differences between the baseline characteristics of the IG and CG. In accordance with the CONSORT statement both “intention-to-treat” and “per-protocol” (i.e., using the sample of participants who adequately adhered to the intervention protocol by completing at least five out of the 21 sessions) analyses will be conducted for comparison. We will also conduct sensitivity analyses to investigate whether missing data, baseline differences, or the total number of listening sessions has any impact on the outcomes.

For analysing the effect of the intervention on the primary outcome (i.e., individual goal attainment), caregivers’ GAS-ratings will be averaged for each participant across all specified goals. Then, we will use t-tests to compare the average goal attainment between members of the IG and CG. In the case of baseline differences in the IG and CG, we will conduct regression analyses to assess the impact of the intervention on individual goal attainment while statistically controlling for the relevant variables. In addition, we will calculate and compare the proportion of participants who completely or partially attained their goals, maintained the initial state, or reported mild/ severe deterioration of their goals in each group.

Secondary outcomes (including data from the daily assessments) will be analysed using multilevel models with two levels, with assessment points (level 1) nested in participants (level 2). Average treatment effects will be analysed by including group membership as a level-2 predictor (0 = CG, 1 = IG). Person-level moderator variables will be included only if necessary, in accordance with the results of the sensitivity analyses. For outcomes that will be assessed for people with dementia as well as for their caregivers (e.g., physiological stress), three-level multilevel models will be used to account for within and between dyadic effects. Assessment points (level 1) will be nested in participants (level 2), and participants will be nested within dyads (level 3). Full maximum likelihood estimation will be used to account for any missing data.

To analyse the data from the behavioural observations, we will compute frequency scores for time units 1–5 (i.e., 20 min of behavioural observation bevor IML; pre-intervention period in the IG), 6–10 (i.e., 20 min of IML in the IG) and 11–15 (i.e., 20 min of behavioural observation after IML; post-intervention period in the IG). These frequency scores will then be compared within and between both groups (IG vs. CG).

Finally, to assess feasibility and acceptance, we will use a qualitative content analysis to analyse the content of the semi-structured interviews. Video recordings are analysed qualitatively. We will also examine the descriptive statistics of the quantitative measures of playlist quality, intervention fidelity, user-friendliness of the app and tablet, and overall experience with the intervention.

### Ethical approval

Ethical approval was obtained from the Ethics Committee of the Faculty of Social and Behavioural Sciences of the Friedrich Schiller University Jena (committee’s reference number: FSV 22/013). The IML intervention is a non-invasive.

## Discussion

This RCT evaluates the effectiveness of an app-based IML intervention for the home care setting. Due to large and growing population of people with dementia – most of whom are living at home and cared for by family members – finding ways to improve the quality of life of people with dementia and their caregivers represents one of society’s most pressing public health challenges. IML has been shown to be a highly accepted, affordable and effective non-pharmacological intervention for people with dementia in the institutionalized setting. Building on these positive findings, we examine whether the current app-based IML intervention can be successfully implemented in the home care setting and improve the well-being and quality of life of people with dementia and their caregivers. The app is designed to provide an easy, affordable and effective way to construct personally-relevant music playlists and implement IML interventions at home. Integrated tutorials empower family caregivers to successfully implement the intervention and manage arising challenges.

A major strength of the study is that we use a variety of assessment methods and consider the perspectives of people with dementia, their caregivers, as well as external observers and physiological measures of stress. We employ traditional paper-and-pencil questionnaires, app-based questionnaire measures, in-person behavioural observations, video analysis, semi-structured interviews and hair analysis. We consider how IML is related to daily fluctuations as well as longer-term changes. The daily assessments via app help us to avoid retrospective bias and increase the ecological validity of our study. Finally, we consider how IML affects not only scores on standardized measures but also individual goal attainment. Our measures are specifically designed for the target group of people with dementia who have diverse abilities and symptoms, and who may have difficulty communicating. The behavioural observations and measures of acute and chronic stress allow us to assess people with dementia’s well-being without verbal or written communication, and the DeCS coding system [17] was adapted to our non-pharmacological intervention and the observed group of people with dementia. Further strengths of the study include the sufficiently powered sample of dyads and the inclusion of people at all stages and with all types of dementia. However, the study also has limitations: the design does not allow blinding, and the extent to which family caregivers implement the intervention as intended remains to be seen.

The relevance of feasible and effective interventions that improve the quality of life of people with dementia living at home and their caregivers is evident. Our study will demonstrate the extent to which an app-based IML intervention for people with dementia living at home can be delivered by family caregivers and improve the well-being of people with dementia and caregivers, as well as improve the caregiving experience. Insight regarding the acceptance and usability of the app and the tablet will inform further development of the app and other digital assistance technologies. A sustainability concept is being developed for the continuation of IML in practice.

### Supplementary Information

**Supplementary Material 1. **

**Supplementary Material 2. **

## Data Availability

The datasets used and/or analysed during the current study are available from the corresponding author on reasonable request.

## Abbreviations

BPSD: Behavioural and psychological symptoms of dementia
CG: Control group
DeCS: Dementia Coding System
EMA: Ecological Momentary Assessment
GAS: Goal attainment scaling
IG: Intervention group
IML: Individualized music listening
MMSE: Mini-Mental-Status Examination
RCT: Randomized controlled trial
T0: Baseline assessment
T1: Pretest assessment
T2: Posttest assessment
T3: Follow-up
VAS: Visual Analogue Scale
