# Supplementary material for: Study protocol: randomized controlled trial of an individualized music intervention for people with dementia in the home care setting
Source: BMC Psychiatry. 2024 Mar 26;24:230. doi: 10.1186/s12888-024-05697-0 (PMC10967058; doi:10.1186/s12888-024-05697-0)
Supplement: Supplementary file 1 — Supplementary Material 1. [file 12888_2024_5697_MOESM1_ESM.docx]

| **Week** | **1** | | **7** | | **8** | **9** | **10** | **11** | **12** | | **18** |
| --- | --- | --- | --- | --- | --- | --- | --- | --- | --- | --- | --- |
|  | ***Baseline T0*** | ***Pre-test T1*** | | ***Intervention period*** | | | | | | ***Post-test T2*** | ***Follow-up T3*** |
| **Outcomes** | Individual goal established  Person with dementia: well-being, arousal, BPSD  Caregiver’s well-being and arousal  Caregiving experience  Dyadic interaction quality | Person with dementia: well-being, arousal, BPSD  Caregiver’s well-being and arousal  Caregiving experience  Dyadic interaction quality | | *Daily assessments*: well-being and arousal of person with dementia, caregiver’s well-being and arousal, dyadic interaction quality | | | | | | Individual goal attainment  Person with dementia: well-being, arousal, BPSD  Caregiver’s well-being and arousal  Caregiving experience  Dyadic interaction quality | Person with dementia: well-being, arousal, BPSD  Caregiver’s well-being and arousal  Caregiving experience  Dyadic interaction quality |
|  |  |  |  | Intervention group: current mood before and after listening, immediate reactions to individualized music listening via app; quantitative indicators of intervention fidelity | | | | | |  |  |
|  |  |  |  | *Home visit*  Observation of behaviour  Person with dementia: well-being and quality of life  Dyadic interaction quality rated by a project member |  | *Home visit in week 3 or 4*  Observation of  behaviour  Person with dementia: well-being and quality of life  Dyadic interaction quality rated by a project member | |  | *Home visit*  Observation  of behaviour  Person with dementia: well-being and quality of life  Dyadic interaction quality rated by a project member |  |  |
|  |  |  |  | Physiological stress of the dyad: hair cortisol, heart rate variability |  | Physiological stress of the dyad:  heart rate variability | |  | Physiological stress of the dyad: hair cortisol, heart rate variability |  |  |
|  |  |  |  |  |  |  | |  | Intervention experiences, fidelity and acceptance |  |  |
